# Supplementary material for: Comparative genomics provides new insights into the diversity, physiology, and sexuality of the only industrially exploited tremellomycete: Phaffia rhodozyma
Source: BMC Genomics. 2016 Nov 9;17:901. doi: 10.1186/s12864-016-3244-7 (PMC5103461; doi:10.1186/s12864-016-3244-7)
Supplement: Additional file 6: — List of orphan genes with links to PFAM (related to Additional file 1: Table S1). (ZIP 1428 kb) [file 12864_2016_3244_MOESM6_ESM.zip › BLAST_HTML_FTR/G05766_P.html]

BLAST Search Results


```
BLASTP 2.2.27+


Reference:
Stephen F. Altschul, Thomas L. Madden, Alejandro A. Schäffer,
Jinghui Zhang, Zheng Zhang, Webb Miller, and David J. Lipman (1997),
"Gapped BLAST and PSI-BLAST: a new generation of protein database
search programs", Nucleic Acids Res. 25:3389-3402.


Reference for
composition-based statistics:
Alejandro A. Schäffer, L. Aravind, Thomas L. Madden, Sergei
Shavirin, John L. Spouge, Yuri I. Wolf, Eugene V. Koonin, and
Stephen F. Altschul (2001), "Improving the accuracy of PSI-BLAST
protein database searches with composition-based statistics and
other refinements", Nucleic Acids Res. 29:2994-3005.


Database: nr
           71,551,133 sequences; 26,053,659,533 total letters


Query= G05766_P

Length=944
                                                                      Score     E
Sequences producing significant alignments:                          (Bits)  Value

emb|CED84171.1|  hypothetical protein [Xanthophyllomyces dendrorh...  1874    0.0  


 >emb|CED84171.1| hypothetical protein [Xanthophyllomyces dendrorhous]
Length=991

 Score = 1874 bits (4854),  Expect = 0.0, Method: Compositional matrix adjust.
 Identities = 926/933 (99%), Positives = 929/933 (99%), Gaps = 0/933 (0%)

Query  11   LVLPLASHLPAVLDCDRLEPRGLLSDTLSDVSSIHSSDLTSDDEDRADLVRFGDDLAGST  70
            L L +  HLPAVLDCDRLEPRGLLSDTLSDVSSIHSSDLTSDDEDRADLVRFGDDLAGST
Sbjct  59   LYLLIEFHLPAVLDCDRLEPRGLLSDTLSDVSSIHSSDLTSDDEDRADLVRFGDDLAGST  118

Query  71   WTDLGPNTATTDFLSHAFSSGASSAAGDEFEPDSTPGSSSESGRGDGSLTDDEDDHIDND  130
            WTDLGPNTATTDFLSHAFSSGASSAAGDEFEPDSTPGSSSESGRGDGSLTDDEDDHIDND
Sbjct  119  WTDLGPNTATTDFLSHAFSSGASSAAGDEFEPDSTPGSSSESGRGDGSLTDDEDDHIDND  178

Query  131  RTFSGLGESFADLEPSIPSASSLSTSVATTTISDLTDRQHSNLSLYSKFSNACDIEPPTS  190
            RTFSGLGESFADLEPSIPSASSLSTSVATTTISDLTDRQHSNLSLYSKFSNACDIEPPTS
Sbjct  179  RTFSGLGESFADLEPSIPSASSLSTSVATTTISDLTDRQHSNLSLYSKFSNACDIEPPTS  238

Query  191  VPDMRSSVETTSFRRPVKEPVSSHFQLAYPDPTSVSSTFSSPGSSSTLSKSKHECFPSSF  250
            VPDMRSSVETTSFRRPVKEPVSSHFQLAYPDPTSVSSTFSSPGSSSTLSKSKHECFPSSF
Sbjct  239  VPDMRSSVETTSFRRPVKEPVSSHFQLAYPDPTSVSSTFSSPGSSSTLSKSKHECFPSSF  298

Query  251  SQAPSFRETGTGGLGSSHSRSAAGKQATSSFAPASAMIVPTTVKERLTESHSKQEMDGQS  310
            SQAPSFRETGTGGLGSSHSRSAAGKQATSSFAPASAMIVPTTVKERLTESHSKQEMDGQS
Sbjct  299  SQAPSFRETGTGGLGSSHSRSAAGKQATSSFAPASAMIVPTTVKERLTESHSKQEMDGQS  358

Query  311  SRLTNSSYSTDRWTVFDTPSMSRSLQSFPSLVQSTVNLPADVSHPIRILLVGSSGPSNQR  370
            SRLTNSSYSTDRWTVFDTPSMSRSLQSFPSLVQSTVNLPADVSHPIRILLVGSSGPSNQR
Sbjct  359  SRLTNSSYSTDRWTVFDTPSMSRSLQSFPSLVQSTVNLPADVSHPIRILLVGSSGPSNQR  418

Query  371  QTIVQKLLRLVYIILPETAASDPIVLKEFDVLGRSESTRTISDLREPFKRERKTSSLGGR  430
            QTIVQKLLRLVYIILPETAASDPIVLKEFDVLGRSESTRTISDLREPFKRERKTSSLGGR
Sbjct  419  QTIVQKLLRLVYIILPETAASDPIVLKEFDVLGRSESTRTISDLREPFKRERKTSSLGGR  478

Query  431  QPESIDRLTGPKGSLVVEVVDLTINEDDDWVSASEDVRAHLRSLRTSPSITTLVTNLVDP  490
            QPESIDRLTGPKGSLV+EVVDLTINEDDDWVSASEDVRAHLRSLRTSPSITTLVTNLVDP
Sbjct  479  QPESIDRLTGPKGSLVIEVVDLTINEDDDWVSASEDVRAHLRSLRTSPSITTLVTNLVDP  538

Query  491  SQRHRSNLELSSILSSSDRTNMHTIILPVEISASFNPGADLSRDNLIAKSKANTENRVWR  550
            SQRHRSNLELSSILSSSDRTNMHTI+LPVEISASFNPGADLSRDNLIAKSKANTENRVWR
Sbjct  539  SQRHRSNLELSSILSSSDRTNMHTIVLPVEISASFNPGADLSRDNLIAKSKANTENRVWR  598

Query  551  YEDFVGLSGEKDALEVWKGLSLESLTTETVSLARGQTEQEAEEYMFRLSVDWKRLRACVR  610
            YEDFVGLSGEKDALEVWKGLSLESLTTETVSLARGQTEQEAEEYMFRLSVDWKRLRACVR
Sbjct  599  YEDFVGLSGEKDALEVWKGLSLESLTTETVSLARGQTEQEAEEYMFRLSVDWKRLRACVR  658

Query  611  RYVIAVSSLATALLMLLACLDYALEAFEQTSLNPAINVTRSFIKEASIPLSVSPMTARVQ  670
            RYVIAVSSLATALLMLLACLDYALEAFEQTSLNPAINVTRSFIKEASIPLSVSPMTARVQ
Sbjct  659  RYVIAVSSLATALLMLLACLDYALEAFEQTSLNPAINVTRSFIKEASIPLSVSPMTARVQ  718

Query  671  RFSPNLVWTTSVQSAVATPVYRAASASASARGAVPSETRNLITSEAALREESGSLVTKLT  730
            RFSPNLVWTTSVQSAVATPVYRAASASASARGAVPSETRNLITSEAALREESGSLVTKLT
Sbjct  719  RFSPNLVWTTSVQSAVATPVYRAASASASARGAVPSETRNLITSEAALREESGSLVTKLT  778

Query  731  TIKVPSEISVWSVKDRSVMAWKTRVGLPSSLPRRGDFCVNRSVPSFQHVPLNQSSSVKQQ  790
            TIKVPSEISVWSVKDRSVMAWKTRVGLPSSLPRRGDFCVNRSVPSFQHVPLNQSSSVKQQ
Sbjct  779  TIKVPSEISVWSVKDRSVMAWKTRVGLPSSLPRRGDFCVNRSVPSFQHVPLNQSSSVKQQ  838

Query  791  VWKLSEYVHPTVFALPTSLPDSFPLRRLTLDAEMFNPVIVSVQKAYCQTKNVVHALVARS  850
            VWKLSEYVHPTVFALPTSLPDSFPLRRLTLDAEMFNPVIVSVQKAYCQTKNVVHALVARS
Sbjct  839  VWKLSEYVHPTVFALPTSLPDSFPLRRLTLDAEMFNPVIVSVQKAYCQTKNVVHALVARS  898

Query  851  NPSAQSAYNTLHNLRDTVLDLIWPPSPIVHELVEYAVNKVNQILVEVWMRADRSLRRGKM  910
            NPSAQSAYNTLHNLRDTVLDLIWPPSPIVHELVEYAVNKVNQILVEVWMRADRSLRRGKM
Sbjct  899  NPSAQSAYNTLHNLRDTVLDLIWPPSPIVHELVEYAVNKVNQILVEVWMRADRSLRRGKM  958

Query  911  MTRDAFEEWKQRKEARRAKKPWARIKAILARRG  943
            MTRDAFEEWKQRKEARRAKKPWARIKAILARRG
Sbjct  959  MTRDAFEEWKQRKEARRAKKPWARIKAILARRG  991


Lambda      K        H        a         alpha
   0.314    0.127    0.360    0.792     4.96 

Gapped
Lambda      K        H        a         alpha    sigma
   0.267   0.0410    0.140     1.90     42.6     43.6 

Effective search space used: 11380064934960


  Database: nr
    Posted date:  Sep 23, 2015 12:05 AM
  Number of letters in database: 26,053,659,533
  Number of sequences in database:  71,551,133


Matrix: BLOSUM62
Gap Penalties: Existence: 11, Extension: 1
Neighboring words threshold: 11
Window for multiple hits: 40
```
